# A Gravidade da Doença Afeta os Parâmetros de Repolarização Ventricular em Pacientes com COVID-19

**DOI:** 10.36660/abc.20200482

**Published:** 2020-11-01

**Authors:** Mevlut Koc, Hilmi Erdem Sumbul, Erdinc Gulumsek, Hasan Koca, Yurdaer Bulut, Emre Karakoc, Tuba Turunc, Edip Bayrak, Huseyin Ali Ozturk, Muhammed Zubeyir Aslan, Abdullah Orhan Demirtas, Yahya Kemal Icen

**Affiliations:** 1 Adana Health Practice and Research Center Department of Cardiology Adana Turquia Adana Health Practice and Research Center - Department of Cardiology, Adana - Turquia; 2 Adana Health Practice and Research Center Department of Internal Medicine Adana Turquia Adana Health Practice and Research Center - Department of Internal Medicine, Adana - Turquia; 3 Cukurova University Faculty of Medicine Department of Internal Medicine Adana Turquia Cukurova University - Faculty of Medicine - Department of Internal Medicine, Adana - Turquia; 4 Adana Health Practice and Research Center Department of Infectious Disease Adana Turquia Adana Health Practice and Research Center - Department of Infectious Disease, Adana - Turquia

**Keywords:** COVID-19/complicações, Betacoronavírus, Doenças Cardiovasculares, Diabetes Melitus, Hipertensão, Pneumonia, Estudo Comparativo

## Abstract

**Fundamento:**

Não há estudos avaliando o intervalo T_pico_-T_fim_ (Tpe), a relação Tpe/QT e a relação Tpe/QTc para avaliar arritmias cardíacas em pacientes com COVID-19.

**Objetivo:**

Visamos investigar se há alterações nos intervalos QT, QTc e Tpe e nas relações Tpe/QT e Tpe/QTc em pacientes com COVID-19.

**Métodos:**

O estudo incluiu 90 pacientes com infecção por COVID-19 e 30 controles saudáveis pareados por sexo e idade. Foram aferidos os intervalos QT, QTc e Tpe e as relações Tpe/QT e Tpe/QTc. Os participantes incluídos no estudo foram divididos nos seguintes 4 grupos: controles saudáveis (grupo I), pacientes com COVID-19 sem pneumonia (grupo II), pacientes com COVID-19 e pneumonia leve (grupo III) e pacientes com COVID-19 e pneumonia grave (grupo IV). Significância estatística foi definida por valor p < 0,05.

**Resultados:**

Verificou-se que a frequência cardíaca basal, a presença de hipertensão e diabetes, a contagem de leucócitos, o nitrogênio ureico no sangue, a creatinina, o potássio, o aspartato aminotransferase, a alanina aminotransferase, o NT-proBNP, a proteína C reativa de alta sensibilidade, o dímero-D, a TncI-as, o intervalo Tpe, a relação Tpe/QT e a relação Tpe/QTc aumentaram do grupo I para o grupo IV e foram significativamente mais altos em todos os pacientes do grupo IV (p < 0,05). A pressão arterial sistólica, a hemoglobina e os níveis de cálcio eram menores no grupo IV e significativamente menores em comparação com os demais grupos (< 0,05). Os intervalos QT e QTc eram semelhantes entre grupos. Determinou-se que os níveis elevados de frequência cardíaca, cálcio, dímero-D, NT-proBNP e PCR-as eram significativamente relacionados a Tpe, Tpe/QT e Tpe/QTc.

**Conclusões:**

Em pacientes com COVID-19 e pneumonia grave, o intervalo Tpe, a relação Tpe/QT e a relação Tpe/QTc, que estão entre os parâmetros de repolarização ventricular, foram aumentados, sem prolongação dos intervalos QT e QTc. A partir deste estudo, não podemos definitivamente concluir que as alterações eletrocardiográficas observadas estão diretamente relacionadas à infecção por COVID-19 ou à inflamação, mas sim associadas a cenários graves de COVID-19, que podem envolver outras causas de inflamação e comorbidades.

## Introdução

Durante os últimos meses de 2019, surgiu uma nova pandemia causada pela síndrome respiratória aguda grave coronavírus 2 (SARS-CoV-2), e seus efeitos ainda estão em andamento. Essa doença, denominada doença coronavírus 2019 (COVID-19), afeta principalmente o trato respiratório, mas tem uma taxa significativa (12% a 28%) de envolvimento cardíaco.^1^^–^^4^ Níveis aumentados de troponina T cardíaca (TnTc), troponina cardíaca I (TncI), e de TncI e TnTc de alta sensibilidade (TncI-as e TnTc-as)^1^^,^^2^^,^^4^ e NT-proBNP^5^ foram encontrados em pacientes com envolvimento cardíaco. A mortalidade aumenta nos pacientes com envolvimento cardíaco.^1^^,^^6^^–^^8^ O envolvimento cardíaco é multifatorial em pacientes com COVID-19.^1^^,^^4^^,^^9^^–^^16^ Uma vez que o envolvimento cardíaco está associado à mortalidade, é possível predizer um aumento da mortalidade devido à arritmia nestes pacientes. De fato, os pacientes com COVID-19 apresentaram arritmias fatais.^1^^–^^3^^,^^9^ Apesar disso, nenhum parâmetro ou classificação clara foi relatada para fornecer informações sobre a frequência das arritmias ou para predizê-las nesses pacientes. Só foi recomendado medir o QT e o QT corrigido (QTc) com antecedência, a fim de reduzir eventos arrítmicos fatais antes de iniciar a hidroxicloroquina e a azitromicina que têm sido usadas na profilaxia e tratamento de COVID-19.^17^

A repolarização ventricular prolongada ou comprometida está associada a arritmias potencialmente fatais, como a taquicardia ventricular (TV) e a fibrilação ventricular (FV). Existem muitos parâmetros de eletrocardiograma (ECG) relacionados à despolarização e à repolarização ventricular. Os parâmetros utilizados na prática clínica são os intervalos QT e QTc, a dispersão de QT e de QTc e o intervalo T_pico_-T_fim_ (Tpe). As relações Tpe/QT e Tpe/QTc obtidas a partir destes parâmetros estão associadas à dispersão transmural ventricular durante a repolarização.^18^ O intervalo Tpe elevado indica disseminação anormal na repolarização ventricular e está associado ao risco aumentado de arritmia ventricular.^19^ Até onde sabemos, não há estudos sobre o QT, o QTc, o intervalo Tpe, a relação Tpe/QT e a relação Tpe/QTc com respeito ao efeito do COVID-19 nos parâmetros de repolarização ventricular. Portanto, o objetivo do nosso estudo foi o de investigar se existem alterações no QT, no QTc, no intervalo Tpe, na relação Tpe/QT e na relação Tpe/QTc em pacientes com COVID-19.

## Materiais e Métodos

Foram examinados retrospectivamente 120 pacientes com diagnóstico de COVID-19, internados em terapia intensiva, serviço de internação ou clínicas de pandemia COVID-19, entre 15 de março e 20 de abril de 2020 e submetidos ao ECG de admissão. Após a aplicação dos critérios de exclusão, o estudo incluiu 30 pacientes com COVID-19 e pneumonia grave (grupo IV, 20 homens e 10 mulheres, idade média 61,2 ± 10,1 anos), 30 pacientes com COVID-19 e pneumonia leve (grupo III, 18 homens e 12 mulheres, idade média 64,8 ± 12,3 anos), 30 pacientes com COVID-19 sem pneumonia (grupo II, 19 homens e 11 mulheres, idade média 65,2 ± 14,2 anos) e 30 controles saudáveis (17 homens e 13 mulheres, idade média 63,5 ± 13,5 anos). Para os pacientes com COVID-19 examinados neste estudo, foram considerados como critérios de exclusão os seguintes fatores: grupo de idade pediátrica (< 18 anos); ausência de medição de Tpe e QTc; doença arterial coronariana ou síndrome coronariana aguda conhecidas; doença cardíaca valvar leve a avançada; insuficiência cardíaca sistólica; qualquer tratamento médico conhecido por prolongar ou encurtar os intervalos QT ou QTc e histórico pessoal ou familiar de síncope ou parada súbita cardíaca. O estudo foi realizado de acordo com a Declaração de Helsinque e foi aprovado pelo comitê de ética local.

Os parâmetros demográficos, clínicos e bioquímicos e o ECG de 12 derivações de todos os pacientes foram obtidos de seus prontuários. Os dados demográficos de todos os pacientes como sexo, frequência cardíaca (FC) basal, pressão arterial sistólica (PAS) e pressão arterial diastólica (PAD), foram obtidos dos arquivos. Usando parâmetros bioquímicos de rotina, contagem de leucócitos, hemograma, nível sanguíneo de glicose, testes de função renal, aspartato aminotransferase (AST), alanina aminotransferase (ALT), nível sérico de cálcio, colesterol de lipoproteína de baixa densidade (LDL), proteína C reativa de alta sensibilidade (PCR-as), dímero-D, N-terminal do peptídeo natriurético cerebral tipo B (NT-pro-BNP) e TncI-as foram registrados.

### Avaliações de Eletrocardiograma de 12 Derivações

ECG de 12 derivações, realizados usando uma máquina de ECG MAC 2000 (GE medical systems information technologies, Inc., WI, EUA), em ritmo sinusal, com velocidade de 25 mm/seg e calibração padrão de 1 mv/10 mm, foram obtidos dos arquivos para todos os indivíduos. Para o intervalo QT, foi calculado o tempo do começo da onda QRS até o ponto onde a onda T se fundia com a linha isoelétrica. *QTc* foi calculado utilizando-se a fórmula de Bazett (*QTc* = *QTc* / √*R* - *R*). O limite superior do normal para QTc foi aceito como 450 e 460 ms para homens e mulheres, respectivamente.^20^ O intervalo Tpe foi definido como o tempo do pico da onda T até o ponto onde a onda T se junta e termina com a linha isoelétrica. As medições foram feitas principalmente a partir de V5. Nos casos em que V5 não era adequado para medição (amplitude < 1,5 mm), as medições foram feitas de V4 ou V6.^21^ As relações *Tpe/QT* e *Tpe/QTc* foram calculadas de acordo com essas medições. Todos os exames de ECG em ritmo sinusal foram avaliados por dois cardiologistas com pelo menos 5 anos de experiência em eletrofisiologia, que avaliam ≥ 2.000 pacientes com arritmias anualmente e que não conheciam o paciente ou a clínica.

### Análise Estatística

O teste de Shapiro-Wilk foi usado para distribuição normal das variáveis contínuas. As variáveis contínuas dos dados dos grupos foram indicadas como média ± desvio padrão ou mediana e intervalo interquartil. As variáveis categóricas foram especificadas como números e porcentagens. As variáveis contínuas que apresentavam distribuição normal foram comparadas pelo teste ANOVA unidirecional, enquanto o teste de Kruskal-Wallis foi usado para comparar amostras sem distribuição normal. Para os dados com distribuição normal, os testes de Scheffe e Games-Howell foram usados para comparações múltiplas de grupos, em relação à homogeneidade das variâncias. Para dados não normalmente distribuídos, o teste U Mann-Whitney ajustado de Bonferroni foi usado para comparações múltiplas entre grupos. Foi usado o teste de qui-quadrado para comparar as variáveis categóricas. As análises de correlação de Pearson e Spearman foram realizadas para determinar os parâmetros relacionados ao intervalo Tpe e as relações de Tpe/QT e Tpe/QTc. Foi realizada a análise de regressão linear para os parâmetros que estavam mais relacionados ao intervalo Tpe e às relações Tpe/QT e Tpe/QTc na análise univariada. Para evitar problemas de multicolinearidade, cada parâmetro de repolarização ventricular foi analisado separadamente em modelos diferentes. Todos os modelos foram ajustados por sexo, idade e fatores de risco cardiovascular. O coeficiente kappa foi utilizado para avaliar a variabilidade interobservador e intraobservador de todas as medidas eletrocardiográficas.

## Resultados

Conforme previamente descrito, os dados do estudo foram divididos em 4 grupos e comparados. As medidas eletrocardiográficas foram obtidas com sucesso em todos os pacientes incluídos no estudo. Os valores do kappa de Cohen que avaliam a variabilidade interobservador e intraobservador foram superiores a 90% para todos os critérios do ECG.

### Dados Demográficos e Clínicos dos Grupos do Estudo

Quando os dados demográficos foram comparados de acordo com os grupos do estudo, a distribuição de idade e sexo foi semelhante entre os grupos. A frequência de hipertensão e diabetes mellitus era mais alta no grupo IV. Entre os parâmetros clínicos, foi demonstrado que os valores de PAS e PAD foram os mais baixos nos pacientes do grupo IV e eram significativamente menores em comparação com os demais grupos (Tabela 1). Também foi demonstrado que o valor basal da FC aumentou do grupo I para o grupo IV e os valores foram significativamente maiores nos pacientes do grupo IV em comparação com os demais grupos (Tabela 1). A PAS, a PAD, e os valores basais de FC dos grupos I, II, e III eram semelhantes (Tabela 1).

**Tabela 1:** Achados clínicos demográficos e laboratoriais de acordo com grupo do estudo

Variável	Grupo I n=30	Grupo II n=30	Grupo III n=30	Grupo IV n=30	p
Idade (anos)	63,5 ± 13,5	65,2 ± 14,2	64,8 ± 12,3	61,2 ± 10,1	0,627
Sexo (masculino/feminino)	17/13	19/11	18/12	20/10	0,506
Hipertensão, n (%)	0 (0%)	7 (23%)	15 (50%)	17 (57%)	<0,001
Diabetes mellitus, n (%)	0 (0%)	6 (20%)	7 (23%)	11(38%)	<0,001
Tabagismo atual, n (%)	0 (0%)	14 (47%)	15 (50%)	12(40%)	0,425
PAS (mmHg)	125 ± 11α	130 ± 10,1β	136 ± 14⁑	108 ± 30	<0,001
PAD (mmHg)	76,9 ± 4,8α	79,9 ± 7,6β	80,2 ± 7,5⁑	62,3 ± 23,1	<0,001
Pulso (bpm)	67 ± 8,2α	68 ± 9,1β	75,6 ± 12,1⁑	89,6 ± 19,5	<0,001
Contagem de leucócitos (uL)	9039 ± 1188α,¥	1097 ± 1516β,Δ	1277 ± 1484⁑	1906 ± 2698	<0,001
Hemoglobina (gr/dL)	13,3 ± 1,05α,¥	13,4 ± 1,58β,Δ	12,7 ± 0,81⁑	10,6 ± 0,74	<0,001
Glicose (mg/dL)	105 ± 13	138 ± 13	141 ± 11	138 ± 12	0,172
Nitrogênio ureico no sangue (mg/dL)	25,3 ± 6,7α	28,1 ± 8,8β	37,2 ± 25⁑	66,9 ± 80	0,001
Creatinina (mg/dL)	0,60 ± 0,07α	0,62 ± 0,07β	0,67 ± 0,18⁑	1,08 ± 0,80	<0,001
Sódio (mEq/L)	140 ± 4,0	140 ± 3,7	140 ± 6,8	140 ± 2,5	0,986
Potássio (mEq/L)	4,31 ± 0,33α	4,34 ± 0,34β	4,30 ± 0,68⁑	4,74 ± 0,58	0,002
Cálcio (mg/dL)	9,45 ± 0,50α,¥	9,47 ± 0,56β,Δ	8,38 ± 0,82⁑	7,99 ± 1,20	<0,001
AST (u/L)	28 (26–29)α,¥	28 (28–29)β,Δ	47 (43–49)⁑	50 (44–56)	<0,001
ALT (u/L)	27 (23–27)α,¥	26 (23–27)β,Δ	34 (33–27)⁑	37 (36–40)	<0,001
Colesterol LDL (mg/dL)	117 ± 25	115 ± 25	117 ± 24	113 ± 25	0,911
PCR-as (mg/dL)	1,2 (1,0–1,4)α	1,2 (1,0–1,4)β	2,1 (1,5–3,1)⁑	17 (11–22)	<0,001
Dímero-D (ng/mL)	4 (3–36)α,¥	4 (4–35)β,Δ	499 (34–725)⁑	750 (499–1550)	<0,001
NT-proBNP (pg/mL)	23 (10–33)α	21 (11–34)β	100 (41–111)⁑	123 (110–567)	0,033
TncI-as (ng/L)	5 (3–13)α	5 (3–14)β	16 (14–30)⁑	20 (14–156)	0,005

Os valores são apresentados como média ± desvio padrão, mediana e intervalo interquartil ou n (%). ALT: alanina aminotransferase; AST: aspartato aminotransferase; LDL: lipoproteína de baixa densidade; NT-proBNP: N-terminal do peptídeo natriurético cerebral tipo B; PAD: pressão arterial diastólica; PAS: pressão arterial sistólica, PCR-as: proteína C reativa de alta sensibilidade; TncI-as: troponina cardíaca I de alta sensibilidade. Grupo I: controles saudáveis, grupo II: pacientes com COVID-19 sem pneumonia, grupo III: pacientes com COVID-19 e pneumonia leve, grupo IV: pacientes com COVID-19 e pneumonia grave.

α = diferença significativa entre grupo I e grupo IV (p < 0,05).

¥ = diferença significativa entre grupo I e grupo III (p < 0,05).

▶ = diferença significativa entre grupo I e grupo II (p < 0,05).

β = diferença significativa entre grupo II e grupo IV (p < 0,05).

Δ = diferença significativa entre grupo II e grupo III (p < 0,05).

⁑ = diferença significativa entre grupo III e grupo IV (p < 0,05).

### Dados Laboratoriais dos Grupos do Estudo

Os parâmetros laboratoriais, como a contagem de leucócitos, o nitrogênio ureico no sangue, creatinina, potássio, AST, ALT, PCR-as, dímero-D, NT-proBNP e TncI-as aumentaram do grupo I para o grupo IV e foram significativamente mais altos nos pacientes do grupo IV em comparação com os demais grupos (Tabela 1). Além disso, a contagem de leucócitos, os níveis de AST, ALT e dímero-D foram significativamente maiores em comparação com o grupo I e o grupo II. Foi determinado que os níveis de hemoglobina e de cálcio diminuíram do grupo I para o grupo IV e foram significativamente menores nos pacientes do grupo IV em comparação com os demais grupos. Também foram menores no grupo III do que nos grupos I e II (Tabela 1).

### Dados Eletrocardiográficos dos Grupos do Estudo

Quando os dados de ECG foram comparados de acordo com os grupos do estudo, os intervalos QT e QTc foram semelhantes em todos os grupos (Tabela 2). Apenas 1 paciente apresentou QTc > 500 ms e 1 paciente apresentou QTc > 460 ms. Os valores de QTc de todos os outros pacientes eram normais. O intervalo Tpe e as relações Tpe/QT e Tpe/QTc aumentaram do grupo I para o grupo IV e foram significativamente maiores em todos os pacientes do grupo IV em comparação com os demais grupos (Tabela 2).

**Tabela 2:** Comparação de achados de despolarização e repolarização ventriculares de acordo com grupo do estudo

Variável	Grupo I n=30	Grupo II n=30	Grupo III n=30	Grupo IV n=30	p
Intervalo QT, tempo (ms)	367 ± 49	380 ± 21	381 ± 24	382 ± 51	0,338
Intervalo QTc, tempo (ms)	405 ± 23	406 ± 34 β, Δ	406 ± 15	407 ± 16	0,989
Intervalo Tpe, tempo (ms)	70,3 ± 7,1 α	72,7 ± 7,7 β	74,1 ± 8,5⁑	90,1 ± 9,2	<0,001
Relação Tpe/QT	0,186 ± 0,021 α	0,191 ± 0,023 β	0,203 ± 0,051⁑	0,235 ± 0,034	<0,001
Relação Tpe/QTc	0,188 ± 0,022 α	0,186 ± 0,024 β	0,200 ± 0,018⁑	0,216 ± 0,029	<0,001

Os valores são apresentados como média ± desvio padrão ou n (%).

Grupo I: controles saudáveis, grupo II: pacientes com COVID-19 sem pneumonia, grupo III: pacientes com COVID-19 e pneumonia leve, grupo IV: pacientes com COVID-19 e pneumonia grave.

α = diferença significativa entre grupo I e grupo IV (p < 0,05).

¥ = diferença significativa entre grupo I e grupo III (p < 0,05).

▶ = diferença significativa entre grupo I e grupo II p<0.05).

β = diferença significativa entre grupo II e grupo IV (p < 0,05).

Δ = diferença significativa entre grupo II e grupo III (p < 0,05).

⁑ = diferença significativa entre grupo III e grupo IV p<0.05).

### A Determinação dos Parâmetros Relacionados ao Intervalo Tpe, à Relação Tpe/QT e à Relação Tpe/QTc

Foi realizada análise de correlação para determinar os parâmetros relacionados ao intervalo Tpe, à relação Tpe/QT e à relação Tpe/QTc. A Tabela 3 resume os parâmetros relacionados ao intervalo Tpe, à relação Tpe/QT e à relação Tpe/QTc na análise de correlação. Foi realizada a análise de regressão linear para determinar os parâmetros significativamente relacionados ao intervalo Tpe, à relação Tpe/QT e à relação Tpe/QTc na análise de correlação (Tabela 4). Como resultado desta análise, foi verificado que os níveis basais de FC, dímero-D e TncI-as eram positiva e significativamente associados ao intervalo Tpe, à relação Tpe/QT e à relação Tpe/QTc. O nível sérico de cálcio foi negativa e significativamente correlacionado com o intervalo Tpe e com a relação Tpe/QTc. Concomitantemente, o NT-proBNP e a relação Tpe/QTc foram positiva e significativamente relacionados. Estatisticamente, a relação mais significativa foi encontrada entre a relação Tpe/QTc e o dímero-D (Tabela 4).

**Tabela 3:** Análises de correlação para parâmetros associados a intervalo Tpe, relação Tpe/QT e relação Tpe/QTc

	Intervalo Tpe		Tpe/QT		Tpe/QTc	
	r	p	r	p	r	p
Pressão arterial sistólica (mmHg)	−0,296	0,001	−0,175	0,056	−0,149	0,105
Pressão arterial diastólica (mmHg)	−0,218	0,017	−0,187	0,040	−0,183	0,046
Pulso (bpm)	0,298	0,001	0,309	0,001	0,125	0,424
Creatinina (mg/dL)	0,279	0,002	0,223	0,014	0,247	0,007
Potássio (mEq/L)	0,274	0,002	0,299	0,001	0,120	0,405
Cálcio (mg/dL)	−0,461	<0,001	−0,241	0,008	−0,287	0,002
PCR-as (mg/dL)	0,245	0,007	0,208	0,023	0,219	0,012
Dímero-D (ng/mL)	0,431	<0,001	0,298	0,001	0,569	<0,001
NT-proBNP (pg/mL)	0,192	0,035	0,190	0,045	0,351	<0,001
TncI-as (ng/L)	0,185	0,042	0,210	0,019	0,255	0,005

NT-proBNP: N-terminal do peptídeo natriurético cerebral tipo B; PCR-as: proteína C reativa de alta sensibilidade; TncI-as: troponina cardíaca I de alta sensibilidade.

**Tabela 4:** Análise de regressão linear para parâmetros significativamente associados a intervalo Tpe, relação Tpe/QT e relação Tpe/QTc

	Intervalo Tpe		Tpe/QT		Tpe/QTc	
	β	p	β	p	β	p
Pressão arterial sistólica (mmHg)	−0,092	0,236	−0,056	0,530	−0,013	0,865
Pressão arterial diastólica (mmHg)	−0,017	0,827	−0,057	0,536	−0,698	0,487
Pulso (bpm)	0,271	0,001	0,286	0,001	0,205	0,007
Creatinina (mg/dL)	0,143	0,054	0,153	0,074	0,054	0,499
Potássio (mEq/L)	0,105	0,168	0,175	0,158	0,158	0,168
Cálcio (mg/dL)	−0,298	<0,001	−0,103	0,263	−0,241	0,002
PCR-as (mg/dL)	0,078	0,306	0,113	0,188	0,021	0,773
Dímero-D (ng/mL)	0,342	<0,001	0,271	0,002	0,493	<0,001
NT-proBNP (pg/mL)	0,114	0,125	0,055	0,526	0,233	0,001
TncI-as (ng/L)	0,235	0,002	0,205	0,010	0,198	0,012

NT-proBNP: N-terminal do peptídeo natriurético cerebral tipo B; PCR-as: proteína C reativa de alta sensibilidade; TncI-as: troponina cardíaca I de alta sensibilidade. R_ajustado_^2^ para intervalo Tpe, Tpe/QT e Tpe/QTc como 0,426, 0,446 e 0,487, respectivamente.

## Discussão

O achado principal do nosso estudo é que, em pacientes com COVID-19 e pneumonia grave, o intervalo Tpe e as relações Tpe/QT e Tpe/QTc são elevados, sem prolongação dos intervalos QT e QTc. Até onde sabemos, este é o primeiro estudo na literatura a demonstrar aumento do intervalo Tpe e das relações Tpe/QT e Tpe/QTc, que estão entre os parâmetros de repolarização ventricular, em pacientes com COVID-19.

A infecção por COVID-19 envolve principalmente as vias aéreas, mas complicações cardiovasculares significativas também podem ocorrer.^1^^–^^3^^,^^9^^,^^10^ Não seria correto explicar o envolvimento cardíaco ou as complicações que ocorrem nesta doença como um mecanismo único; a lesão cardíaca é considerada multifatorial.^4^ Os possíveis mecanismos para o envolvimento cardíaco podem ser resumidos da maneira seguinte: i) miocardite viral direta como o mecanismo mais comumente considerado;^1^^,^^9^^–^^11^ ii) hipotensão e FC elevada;^3^ iii) hipóxia;^14^ iv) inflamação elevada e liberação de citocina;^14^ v) regulação negativa dos receptores ECA-2;^13^ vi) toxicidade de medicamentos (cloroquina, hidroxicloroquina, eritromicina, etc.)^15^^–^^16^ e vii) aumento da liberação endógena de catecolaminas.^12^ Embora todos estes parâmetros não tenham sido observados no nosso estudo, foram verificados aumentos dos níveis de TncI-as e NT-proBNP, que sugere envolvimento miocárdico; aumento na contagem de leucócitos e PCR-as, evidenciando o processo de inflamação; aumento da FC com diminuição da PAS e da PAD, evidenciando o estado hemodinâmico, evoluindo do grupo controle para o grupo com pneumonia grave, em consonância com a literatura. Além disso, no nosso estudo, foi verificado um aumento no nível do dímero-D em pacientes com maior gravidade da doença.

A mortalidade é paralela ao aumento do envolvimento cardíaco em pacientes com COVID-19.^1^^,^^6^^–^^8^ Assim como nas doenças cardiovasculares, a causa mais comum de mortalidade cardíaca em pacientes com COVID-19 são os eventos arrítmicos. Em muitos estudos, foi relatado que os pacientes com COVID-19 e envolvimento cardíaco têm diferentes frequências e tipos de arritmias cardíacas.^1^^–^^3^^,^^9^ Ainda não existe um mecanismo e uma classificação clara das arritmias para essa doença. Num estudo com 187 pacientes, Guo et al.,^2^ relataram uma taxa de envolvimento cardíaco de 27,8%, TV ou FV sendo presente em 5,9% destes pacientes. Zhou et al.,^3^ relataram que a taxa de envolvimento cardíaco foi de 17% em 191 pacientes, e 1% desses pacientes apresentou FC > 125 bpm. Shi et al.,^1^ relataram que a taxa de envolvimento cardíaco foi de 19,7% em 416 pacientes, a depressão do segmento ST sendo encontrada em 0,7% desses pacientes. Wang et al.,^9^ relataram uma frequência de 16,7% de eventos arrítmicos, em 118 pacientes.

O mecanismo mais importante da fisiopatologia da arritmia ventricular em pacientes com infecção por COVID-19 é semelhante ao das arritmias em pacientes com miocardite aguda.^1^^,^^9^^–^^11^ Assim como na miocardite aguda, os motivos mais importantes das arritmias são o aumento da TncI-as e a diminuição da função ventricular esquerda, devido ao aumento do dano miocárdico durante o período agudo, bem como a fibrose atrial e ventricular ocorrendo no período tardio.^22^ Em estudos realizados em pacientes com miocardite aguda em anos prévios, foram verificados aumentos dos intervalos QT, QTc e Tpe e as relações Tpe/QT e Tpe/QTc durante o período agudo.^23^^,^^24^ Até onde sabemos, não existem estudos que pesquisem QT, QTc, o intervalo Tpe, a relação Tpe/QT e a relação Tpe/QTc em casos de miocardite ou envolvimento cardíaco em pacientes com COVID-19. Em nosso estudo, os níveis de TncI-as eram significativamente mais altos nos pacientes com COVID-19 e pneumonia grave. No nosso estudo, não foram realizadas análise e classificação das arritmias porém, os parâmetros de repolarização ventricular dos pacientes, que podem predizer eventos arrítmicos com antecedência, foram avaliados na admissão. Foi verificado que o intervalo Tpe, a relação Tpe/QT e a relação Tpe/QTc, que estão entre os parâmetros de repolarização ventricular, aumentaram com a atividade e gravidade da doença, sendo bem maiores em pacientes com pneumonia grave. Além disso, verificou-se que houve uma associação positiva e significativa entre TncI-as e o intervalo Tpe, a relação Tpe/QT e a relação Tpe/QTc, o que corrobora estudos que mostram que a frequência de arritmias aumentou em pacientes com TnTc-as elevada.

Há muitos parâmetros relacionados à atividade da doença e ao seu prognóstico em pacientes com COVID-19. A maioria dos parâmetros associados à atividade e ao prognóstico também estão associados com o envolvimento cardíaco. No nosso estudo, a atividade da doença foi associada à presença e gravidade da pneumonia. Além disso, os parâmetros comprometidos de repolarização ventricular foram positiva e significativamente relacionados ao aumento de FC,^3^ NT-proBNP,^8^ dímero-D^5^ e TncI-as,^1^^–^^5^ que são intimamente relacionados à atividade da COVID-19 na literatura. Portanto, hipotetizamos que o prolongamento elevado da repolarização miocárdica nos pacientes com COVID-19 pode ser afetado pela atividade da doença e que os eventos arrítmicos nesses pacientes pudessem ser previstos com antecedência.

### Limitações

Há algumas limitações importantes no nosso estudo, incluindo o desenho retrospectivo do estudo e o número de pacientes incluídos. Além disso, os eventos arrítmicos e os parâmetros de acompanhamento clínico não foram avaliados, devido ao número pequeno de pacientes e à falta de acompanhamento clínico. Estudos prospectivos com mais pacientes podem fornecer informações mais significativas. No nosso estudo, os medicamentos e os tratamentos médicos que prolongam o QT foram considerados como critérios de exclusão, mas não foi realizada nenhuma avaliação genética para QT longo ou curto. Esta canalopatia hereditária pode não ser muito significativa, devido à sua raridade. Não foi realizada ressonância magnética para o envolvimento cardíaco ou a miocardite devido à COVID-19. Outra limitação importante do nosso estudo foi a impossibilidade de avaliar os efeitos de drogas, como a hidroxicloroquina e a azitromicina, que são frequentemente utilizadas para tratar COVID-19, na repolarização ventricular.

## Conclusão

Embora o problema principal relacionado à mortalidade e à morbidade em pacientes com COVID-19 seja a doença pulmonar aguda, as evidências disponíveis indicam que um em cada cinco pacientes com COVID-19 apresenta dano miocárdico. O nosso estudo demonstrou que, em adição aos estudos prévios sobre COVID-19 na literatura, o distúrbio da repolarização miocárdica ocorreu em adição ao aumento do dano miocárdico em pacientes com pneumonia grave. Em pacientes com COVID-19, foi verificado que o intervalo Tpe, a relação Tpe/QT e a relação Tpe/QTc, que estão entre os parâmetros de dispersão transmural de repolarização ventricular, eram elevados, sem prolongação dos intervalos QT e QTc. Isso foi mais pronunciado em pacientes com COVID-19 grave e pneumonia grave e pode estar associado ao aumento da inflamação e ao dano miocárdico. Para pacientes com COVID-19, principalmente aqueles com pneumonia grave, deve-se ter em mente que pode ocorrer prolongamento na repolarização ventricular. Neste estudo, não podemos definitivamente concluir que as alterações eletrocardiográficas observadas estão diretamente relacionadas à infecção por COVID-19 ou à inflamação, mas sim associadas a cenários graves de COVID-19, que podem envolver outras causas de inflamação e comorbidades.
