# Supplementary material for: A Gravidade da Doença Afeta os Parâmetros de Repolarização Ventricular em Pacientes com COVID-19
Source: Arq Bras Cardiol. 2020 Nov 1;115(5):907–13. [Article in Portuguese] doi: 10.36660/abc.20200482 (PMC8452192; doi:10.36660/abc.20200482)
Supplement: Translation

# Translation: Disease Severity Affects Ventricular Repolarization Parameters in Patients With COVID-19

**Authors:** Mevlut Koc, Hilmi Erdem Sumbul, Erdinc Gulumsek, Hasan Koca, Yurdaer Bulut, Emre Karakoc, Tuba Turunc, Edip Bayrak, Huseyin Ali Ozturk, Muhammed Zubeyir Aslan, Abdullah Orhan Demirtas, Yahya Kemal Icen

**Affiliations:** 1 Adana Turkey Adana Health Practice and Research Center - Department of Cardiology, Adana - Turkey; 2 Adana Turkey Adana Health Practice and Research Center - Department of Internal Medicine, Adana - Turkey; 3 Adana Turkey Cukurova University - Faculty of Medicine - Department of Internal Medicine, Adana - Turkey; 4 Adana Turkey Adana Health Practice and Research Center - Department of Infectious Disease, Adana - Turkey

**Keywords:** COVID-19/complications, Betacoronavirus, Cardiovascular Diseases, Diabetes Mellitus, Hypertension, Pneumonia, Comparative Study

## Abstract

**Background:**

There is no study evaluating the T_peak_-T_end_ (Tpe) interval, Tpe/QT ratio, and Tpe/QTc ratio to assess cardiac arrhythmias in patients with COVID-19.

**Objective:**

We aimed to examine whether there is a change in QT, QTc, Tpe interval, Tpe/QT ratio, and Tpe/QTc ratio in patients with COVID-19.

**Methods:**

The study included 90 patients with COVID-19 infection and 30 age-and-sex-matched healthy controls. QT, QTc, Tpe interval, Tpe/QT ratio, and Tpe/QTc ratio were measured. The participants included in the study were divided into the following 4 groups: healthy controls (group I), patients with COVID-19 without pneumonia (group II), patients with COVID-19 and mild pneumonia (group III), and patients with COVID-19 and severe pneumonia (group IV). Statistical significance was set at p < 0.05.

**Results:**

It was found that baseline heart rate, presence of hypertension and diabetes, white blood cell count, blood urea nitrogen, creatinine, potassium, aspartate aminotransferase, alanine aminotransferase, NT-proBNP, high sensitive C reactive protein, D-dimer, hs-cTnI, Tpe, Tpe/QT, and Tpe/QTc increased from group I to group IV, and they were significantly higher in all patients in group IV (p < 0.05). Systolic-diastolic blood pressure, hemoglobin, and calcium levels were found to be lowest in group IV and significantly lower than in other groups (< 0.05). QT and QTc intervals were similar between groups. It was determined that increased heart rate, calcium, D-dimer, NT-proBNP and hs-CRP levels were significantly related to Tpe, Tpe/QT, and Tpe/QTc.

**Conclusions:**

In patients with COVID-19 and severe pneumonia, Tpe, Tpe/QT ratio, and Tpe/QTc ratio, which are among ventricular repolarization parameters, were found to be increased, without prolonged QT and QTc intervals. In this study, we cannot definitively conclude that the ECG changes observed are directly related to COVID-19 infection or inflammation, but rather associated with severe COVID-19 scenarios, which might involve other causes of inflammation and comorbidities. (Arq Bras Cardiol. 2020; 115(5):907-913)

## Introduction

In the last months of 2019, a new pandemic caused by severe acute respiratory syndrome coronavirus 2 (SARS-CoV-2) appeared worldwide, and its effects are still ongoing. This disease, called coronavirus disease 2019 (COVID-19), mainly affects the respiratory tract, but it has a significant rate (12% to 28%) of cardiac involvement.^1^^–^^4^ Increased levels of cardiac troponin T (cTnT), cardiac troponin I (cTnI), high sensitivity cTnI and cTnT (hs-cTnI and hs-cTnT),^1^^,^^2^^,^^4^ and NT-proBNP^5^ have been found in patients with cardiac involvement. Mortality increases in patients with cardiac involvement.^1^^,^^6^^–^^8^ Cardiac involvement is multifactorial in patients with COVID-19.^1^^,^^4^^,^^9^^–^^16^ Since cardiac involvement is associated with mortality, an increase in mortality due to arrhythmia can be predicted in these patients. Patients with COVID-19 have been shown to have fatal arrhythmias.^1^^–^^3^^,^^9^ However, no parameter or clear classification has been reported to provide information regarding the frequency of arrhythmia or to predict it these patients. It has only been recommended to measure QT and corrected QT (QTc) in advance, in order to reduce fatal arrhythmic events before starting hydroxychloroquine and azithromycin which have been used in COVID-19 prophylaxis and treatment.^17^

Prolonged or impaired ventricular repolarization is associated with life-threatening arrhythmias such as ventricular tachycardia (VT) and ventricular fibrillation (VF). There are many electrocardiography (ECG) parameters related to impaired ventricular depolarization and repolarization. The parameters used in clinical practice are the QT and QTc intervals, QT and QTc dispersion, and the T_peak_-T_end_ (Tpe) interval. The Tpe/QT and Tpe/QTc ratios obtained from these parameters are associated with ventricular transmural dispersion during repolarization.^18^ Increased Tpe interval indicates abnormal spread in ventricular repolarization, and it is associated with increased risk of ventricular arrhythmia.^19^ To the best of our knowledge, there is no study on QT, QTc, Tpe interval, Tpe/QT ratio, and Tpe/QTc ratio regarding the effect of COVID-19 on ventricular repolarization parameters. Therefore, the aim of our study was to investigate whether there is a change in QT, QTc, Tpe interval, Tpe/QT ratio, and Tpe/QTc ratio in patients with COVID-19.

## Materials and Methods

A total of 120 patients diagnosed with COVID-19, who were admitted to intensive care, inpatient service, and COVID-19 pandemic clinics between March 15 and April 20, 2020 and who underwent admission ECG, were scanned retrospectively. After exclusion criteria were applied, the study included 30 patients with COVID-19 and severe pneumonia (group IV, 20 men and 10 women, mean age 61.2 ± 10.1 years), 30 patients with COVID-19 and mild pneumonia (group III, 18 men and 12 women, mean age 64.8 ± 12.3 years), 30 patients with COVID-19 without pneumonia (group II, 19 men and 11 women, mean age 65.2 ± 14.2 years), and 30 healthy controls (17 men and 13 women, mean age 63.5 ± 13.5 years), who were admitted to the outpatient clinics. In patients with COVID-19 who were scanned in this study, the following factors were considered as exclusion criteria: pediatric age group (< 18 years), failure to perform Tpe and QTc measurements, known coronary artery disease or acute coronary syndrome, mild to advanced valvular heart disease, systolic heart failure, any medical treatment known to prolong or shorten the QT and QTc intervals, and personal or family history of syncope or sudden cardiac arrest. The study was conducted in accordance with the Declaration of Helsinki, and it received approved from the local ethics committee.

Demographic, clinical, and biochemical parameters and 12-lead ECG of all patients were obtained from their files. Demographic data of all patients, sex, baseline heart rate (HR), systolic blood pressure (SBP), and diastolic blood pressure (DBP) were recorded from archived files. Using routine biochemistry parameters, white blood cell (WBC) count, hemogram, blood glucose level, kidney function tests, aspartate aminotransferase (AST), alanine aminotransferase (ALT), serum calcium level, low density lipoprotein (LDL) cholesterol, high sensitive C reactive protein (hs-CRP), D-dimer, N-terminal pro-brain natriuretic peptide (NT-proBNP), and hs-cTnI values were recorded.

### Twelve Lead Electrocardiographic Evaluation

Twelve-lead ECG, carried out by MAC 2000 ECG Machine (GE medical systems information technologies, Inc., WI, USA) in sinus rhythm, 25 mm/sec speed and 1 mv/10 mm standard calibration, was obtained from files for all individuals. For the QT interval, the time from where QRS started to the point where the T wave merges with the isoelectric line was calculated. *QT c* was calculated using the Bazett Formula (*QT c* = *QT* / √*R* - *R*). Upper limit of normal for QTc was accepted as 450 and 460 ms for men and women, respectively.20 Tpe interval was defined as the time from the peak of the T wave to the point where the T wave joins and ends with the isoelectric line. Measurements were made primarily from V5. In cases where V5 was not suitable for measurement (amplitude < 1.5 mm), measurements were made from V4 or V6.21 *Tpe/QT* and *Tpe/QTc* ratios were calculated according to these measurements. All ECG examinations in sinus rhythm were evaluated by two cardiologists with at least 5 years of electrophysiology experience, who evaluates ≥ 2000 arrhythmia patients annually and who were not aware of the patient or clinic.

### Statistical Analysis

Shapiro-Wilk test was used for normal distribution of continuous variables. Continuous variables in group data were indicated with mean ± standard deviation or median and interquartile range. Categorical variables were specified as numbers and percentages. Continuous variables that showed normal distribution were compared using the one-way ANOVA test, whereas the Kruskal-Wallis test was used to compare non-normally distributed samples. For normally distributed data, Scheffe and Games-Howell tests were used for multiple comparisons of groups with respect to homogeneity of variances. For non-normally distributed data, Bonferroni adjusted Mann-Whitney U test was used for multiple comparisons of groups. Chi-square test was used to compare categorical variables. Pearson's and Spearman's correlation analyses were performed to determine parameters related to Tpe interval and Tpe/QT and Tpe/QTc ratios. Linear regression analysis was performed for parameters that were more closely to the Tpe interval and Tpe/QT and Tpe/QTc ratios in univariate analysis. In order to avoid multicollinearity problems, each ventricular repolarization parameter was analyzed separately in different models. All models were adjusted by sex, age, and cardiovascular risk factors. The kappa coefficient was used to evaluate interobserver and intraobserver variability of the all ECG measurements. Statistical significance was set at p < 0.05. All analyses were performed using SPSS 22.0 (Chicago, IL, USA) statistical software package.

## Results

As previously stated, the study data were divided into 4 groups and compared. ECG measurements were successfully obtained from all patients included in the study. Cohen kappa values that evaluate interobserver and intraobserver variability were greater than 90% for all ECG criteria.

### Demographic and Clinical Data of the Study Groups

When demographic data were compared according to study groups, age and sex distribution were similar between groups. Hypertension and diabetes mellitus were more frequent in group IV. Among clinical parameters, it was demonstrated that the SBP and DBP values were lowest in group IV patients, and they were significantly lower than all other groups (Table 1). It was also demonstrated that the baseline HR value increased from group I to group IV, and they were significantly higher in patients in group IV than in all other groups (Table 1). SBP, DBP, and baseline HR values of groups I, II, and III were similar (Table 1).

**Table 1:** Clinical, demographic, and laboratory findings according to study group

Variable	Group I n=30	Group II n=30	Group III n=30	Group IV n=30	p
Age (years)	63.5 ± 13.5	65.2 ± 14.2	64.8 ± 12.3	61.2 ± 10.1	0.627
Sex (male/female)	17/13	19/11	18/12	20/10	0.506
Hypertension, n (%)	0 (0%)	7 (23%)	15 (50%)	17 (57%)	<0.001
Diabetes mellitus, n (%)	0 (0%)	6 (20%)	7 (23%)	11(38%)	<0.001
Current smoker, n (%)	0 (0%)	14 (47%)	15 (50%)	12(40%)	0.425
SBP (mmHg)	125 ± 11α	130 ± 10.1β	136 ± 14⁑	108 ± 30	<0.001
DBP (mmHg)	76.9 ± 4.8α	79.9 ± 7.6β	80.2 ± 7.5⁑	62.3 ± 23.1	<0.001
Pulse (bpm)	67 ± 8.2α	68 ± 9.1β	75.6 ± 12.1⁑	89.6 ± 19.5	<0.001
White blood cell (uL)	9039 ± 1188α,¥	1097 ± 1516β,Δ	1277 ± 1484⁑	1906 ± 2698	<0.001
Hemoglobin (gr/dL)	13.3 ± 1.05α,¥	13.4 ± 1.58β,Δ	12.7 ± 0.81⁑	10.6 ± 0.74	<0.001
Glucose (mg/dL)	105 ± 13	138 ± 13	141 ± 11	138 ± 12	0.172
Blood urea nitrogen (mg/dL)	25.3 ± 6.7α	28.1 ± 8.8β	37.2 ± 25⁑	66.9 ± 80	0.001
Creatinine (mg/dL)	0.60 ± 0.07α	0.62 ± 0.07β	0.67 ± 0.18⁑	1.08 ± 0.80	<0.001
Sodium (mEq/L)	140 ± 4.0	140 ± 3.7	140 ± 6.8	140 ± 2.5	0.986
Potassium (mEq/L)	4.31 ± 0.33α	4.34 ± 0.34β	4.30 ± 0.68⁑	4.74 ± 0.58	0.002
Calcium (mg/dL)	9.45 ± 0.50α,¥	9.47 ± 0.56β,Δ	8.38 ± 0.82⁑	7.99 ± 1.20	<0.001
AST (u/L)	28 (26–29)α,¥	28 (28–29)β,Δ	47 (43–49)⁑	50 (44–56)	<0.001
ALT (u/L)	27 (23–27)α,¥	26 (23–27)β,Δ	34 (33–27)⁑	37 (36–40)	<0.001
LDL cholesterol (mg/dL)	117 ± 25	115 ± 25	117 ± 24	113 ± 25	0.911
hs-CRP (mg/dL)	1.2 (1.0–1.4)α	1.2 (1.0–1.4)β	2.1 (1.5–3.1)⁑	17 (11–22)	<0.001
D-dimer (ng/mL)	4 (3–36)α,¥	4 (4–35)β,Δ	499 (34–725)⁑	750 (499–1550)	<0.001
NT-proBNP (pg/mL)	23 (10–33)α	21 (11–34)β	100 (41–111)⁑	123 (110–567)	0.033
hs-cTnI (ng/L)	5 (3–13)α	5 (3–14)β	16 (14–30)⁑	20 (14–156)	0.005

Values are shown as mean ± standard deviation, median and interquartile range, or n (%). ALT: alanine aminotransferase; AST: aspartate aminotransferase; DBP: diastolic blood pressure; hs-CRP: high sensitive C reactive protein; hs-cTnI: high sensitive cardiac troponin I; LDL: low density lipoprotein; NT-proBNP: N-terminal pro-brain natriuretic peptide; SBP: systolic blood pressure. Group I: Healthy controls, group II: patients with COVID-19 without pneumonia, group III: patients with COVID-19 and mild pneumonia, group IV: patients with COVID-19 and severe pneumonia.

α = significant difference between group I and group IV (p < 0.05).

¥ = significant difference between group I and group III (p < 0.05).

▶ = significant difference between group I and group II (p < 0.05).

β = significant difference between group II and group IV (p < 0.05).

Δ = significant difference between group II and group III (p < 0.05).

⁑ = significant difference between group III and group IV (p < 0.05).

### Laboratory Data of the Study Groups

Laboratory parameters such as WBC, blood urea nitrogen, creatinine, potassium, AST, ALT, hs-CRP, D-dimer, NT-proBNP, and hs-cTnI levels increased from group I to group IV, and they were significantly higher in patients in group IV than in all groups (Table 1). In addition, WBC, AST, ALT and D-dimer levels were significantly higher than group I and group II. It was determined that hemoglobin and calcium levels decreased from group I to group IV, and they were significantly lower in patients in group IV than in all other groups. They were also lower in group III than in groups I and II (Table 1).

### Electrocardiographic Data of Study Groups

When ECG data were compared according to study groups, the QT and QTc intervals were found to be similar across all groups (Table 2). Only 1 patient had QTc > 500 ms, and 1 patient had QTc > 460 ms. The QTc values of all other patients were normal. The Tpe interval and the Tpe/QT and Tpe/QTc ratios increased from group I to group IV, and they were significantly higher in all patients in group IV than in those in all other groups (Table 2).

**Table 2:** Comparison of ventricular depolarization and repolarization findings according to study groups.

Variable	group I n=30	group II n=30	group III n=30	group IV n=30	p
QT interval, time (ms)	367 ± 49	380 ± 21	381 ± 24	382 ± 51	0.338
QTc interval, time (ms)	405 ± 23	406 ± 34 β,Δ	406 ± 15	407 ± 16	0.989
Tpe interval, time (ms)	70.3 ± 7.1α	72.7 ± 7.7β	74.1 ± 8.5⁑	90.1 ± 9.2	<0.001
Tpe/QT ratio	0.186 ± 0.021α	0.191 ± 0.023β	0.203 ± 0.051⁑	0.235 ± 0.034	<0.001
Tpe/QTc ratio	0.188 ± 0.022α	0.186 ± 0.024β	0.200 ± 0.018⁑	0.216 ± 0.029	<0.001

Values are shown as mean ± standard deviation or n (%). Group I: Healthy controls, group II: patients with COVID-19 without pneumonia, group III: patients with COVID-19 and mild pneumonia, group IV: patients with COVID-19 and severe pneumonia.

α = significant difference between group I and group IV (p < 0.05).

¥ = significant difference between group I and group III (p < 0.05).

▶ = significant difference between group I and group II p<0.05).

β = significant difference between group II and group IV (p < 0.05).

Δ = significant difference between group II and group III (p < 0.05).

⁑ = significant difference between group III and group IV p<0.05).

### Determination of Parameters Related to Tpe Interval, Tpe/QT ratio, and Tpe/QTc ratio

Correlation analysis was performed to determine parameters related to Tpe interval, Tpe/QT ratio, and Tpe/QTc ratio. Table 3 summarizes parameters related to Tpe interval, Tpe/QT ratio, and Tpe/QTc ratio in correlation analysis. Linear regression analysis was performed to determine the parameters significantly related to Tpe interval, Tpe/QT ratio, and Tpe/QTc ratio in correlation analysis (Table 4). As a result of this analysis, baseline HR, D-dimer, and hs-cTnI levels were found to be positively and significantly associated with Tpe interval, Tpe/QT ratio, and Tpe/QTc ratio. Serum calcium level was negatively and significantly correlated with Tpe interval and Tpe/QTc ratio. Concomitantly, NT-proBNP and Tpe/QTc ratio were positively and significantly related. Statistically, the most significant relationship was found between Tpe/QTc and D-dimer (Table 4)

**Table 3:** Correlation analyses for parameters associated with Tpe interval, Tpe/QT ratio, and Tpe/QTc ratio

	Tpe interval		Tpe/QT		Tpe/QTc	
	r	p	r	p	r	p
Systolic blood pressure (mmHg)	−0.296	0.001	−0.175	0.056	−0.149	0.105
Diastolic blood pressure (mmHg)	−0.218	0.017	−0.187	0.040	−0.183	0.046
Pulse (bpm)	0.298	0.001	0.309	0.001	0.125	0.424
Creatinine (mg/dL)	0.279	0.002	0.223	0.014	0.247	0.007
Potassium (mEq/L)	0.274	0.002	0.299	0.001	0.120	0.405
Calcium (mg/dL)	−0.461	<0.001	−0.241	0.008	−0.287	0.002
hs-CRP (mg/dL)	0.245	0.007	0.208	0.023	0.219	0.012
D-dimer (ng/mL)	0.431	<0.001	0.298	0.001	0.569	<0.001
NT-proBNP (pg/mL)	0.192	0.035	0.190	0.045	0.351	<0.001
hs-cTnI (ng/L)	0.185	0.042	0.210	0.019	0.255	0.005

hs-CRP: High sensitive C reactive protein; hs-cTnI: high sensitive cardiac troponin I; NT-proBNP: N-terminal probrain natriuretic peptide.

**Table 4:** Linear regression analysis for parameters significantly associated with Tpe interval, Tpe/QT ratio, and Tpe/QTc ratio

	Tpe interval		Tpe/QT		Tpe/QTc	
	β	p	β	p	β	p
Systolic blood pressure (mmHg)	−0.092	0.236	−0.056	0.530	−0.013	0.865
Diastolic blood pressure (mmHg)	−0.017	0.827	−0.057	0.536	−0.698	0.487
Pulse (bpm)	0.271	0.001	0.286	0.001	0.205	0.007
Creatinine (mg/dL)	0.143	0.054	0.153	0.074	0.054	0.499
Potassium (mEq/L)	0.105	0.168	0.175	0.158	0.158	0.168
Calcium (mg/dL)	−0.298	<0.001	−0.103	0.263	−0.241	0.002
hs-CRP (mg/dL)	0.078	0.306	0.113	0.188	0.021	0.773
D-dimer (ng/mL)	0.342	<0.001	0.271	0.002	0.493	<0.001
NT-proBNP (pg/mL)	0.114	0.125	0.055	0.526	0.233	0.001
hs-cTnI (ng/L)	0.235	0.002	0.205	0.010	0.198	0.012

hs-CRP: High sensitive C reactive protein; hs-cTnI: high sensitive cardiac troponin I; NT-proBNP: N-terminal probrain natriuretic peptide. R_adjusted_^2^ for Tpe interval, Tpe/QT, and Tpe/QTc as 0.426, 0.446, and 0.487, respectively.

## Discussion

The main finding of our study is that, in patients with COVID-19 and severe pneumonia, Tpe interval, Tpe/QT ratio, and Tpe/QTc ratio are increased, without prolonged QT and QTc intervals. To the best of our knowledge, this is the first study in the literature to show increased Tpe interval, Tpe/QT ratio, and Tpe/QTc ratio, which are among ventricular repolarization parameters, in patients with COVID-19.

COVID-19 infection mainly involves the airways, but significant cardiovascular complications can also occur.^1^^–^^3^^,^^9^^,^^10^ It is not correct to explain the cardiac involvement or complications occurring in this disease as a single mechanism, and cardiac injury is considered to be multifactorial.^4^ Possible mechanisms for cardiac involvement can be summarized as follows: i) direct viral myocarditis as the most commonly considered mechanism,^1^^,^^9^^–^^11^ ii) hypotension and increased HR,^3^ iii) hypoxia,^14^ iv) increased inflammation and cytokine release,^14^ v) down regulation of ACE-2 receptors,^13^ vi) drug toxicity (chloroqiune, hydroxychloroqiune, erythromicin, etc.),^15^^–^^16^ and vii) increased endogenous catecholamine release.^12^ Although all these parameters were not observed in our study, there were shown to be increases in hs-cTnI and NT-proBNP levels, which suggests myocardial involvement; increases in WBC and hs-CRP, showing the inflammation process; an increase in HR with a decrease in SBP and DBP, showing hemodynamic status, progressing from the control group to the group with severe pneumonia, in accordance with the literature. In addition, in our study, an increase in D-dimer level was found in patients with increased disease severity.

Mortality increases with increased cardiac involvement in patients with COVID-19.^1^^,^^6^^–^^8^ As with cardiovascular diseases, the most common cause of cardiac mortality in COVID-19 patients is arrhythmic events. In many studies, it has been reported that patients with COVID-19 and cardiac involvement have different frequencies and types of cardiac arrhythmias.^1^^–^^3^^,^^9^ There is still no clear arrhythmia mechanism and classification for this disease. Guo et al.^2^ reported that the rate of cardiac involvement in 187 patients was 27.8% and VT or VF were present in 5.9% of these patients. Zhou et al.^3^ reported that the rate of cardiac involvement was 17% in 191 patients, and 1% of these patients had HR > 125 bpm. Shi et al.^1^ reported that the rate of cardiac involvement was 19.7% in 416 patients, and ST depression was found in 0.7% of these patients. Wang et al.^9^ reported a 16.7% frequency of arrhythmic events in 118 patients.

The most important mechanism in the physiopathology of ventricular arrhythmia in patients with COVID-19 infection is similar to that of arrhythmias in patients with acute myocarditis.^1^^,^^9^^–^^11^ As in acute myocarditis, the most important reasons of arrhythmias are increased hs-cTnI and decreased left ventricular functions, due to increased myocardial damage in the acute period, as well as atrial and ventricular fibrosis occurring in the late period.^22^ In studies conducted in patients with acute myocarditis in previous years, QT, QTc, Tpe intervals, Tpe/QT ratio, and Tpe/QTc ratio were found to be increased in the acute period.^23^^,^^24^ To the best of our knowledge, there is no study researching QT, QTc, Tpe interval, Tpe/QT ratio, and Tpe/QTc ratio in cases of myocarditis or cardiac involvement in patients with COVID-19. In our study, hs-cTnI levels were significantly higher in patients with COVID-19 with severe pneumonia. In our study, analysis and classification of arrhythmias were not performed. However, ventricular repolarization parameters of patients, which can predict arrhythmic events in advance, were evaluated at admission. It was determined that Tpe interval, Tpe/QT ratio, and Tpe/QTc ratio, which are among ventricular repolarization parameters, increased with the activity and severity of the disease, and they were much higher in patients with severe pneumonia. In addition, it was determined that there was a positive and significant relationship between hs-cTnI and Tpe, Tpe/QT ratio, and Tpe/QTc ratio, which supports studies showing that the frequency of arrhythmias increased in patients with high hs-cTnT.

There are many parameters related to disease activity and prognosis in patients with COVID-19. The majority of parameters associated with disease activity and prognosis are also associated with cardiac involvement. In our study, disease activity was associated with the presence and severity of pneumonia. In addition, impaired ventricular repolarization parameters in our study were positively and significantly related to increased HR,^3^ NT-proBNP,^8^ D-dimer,^5^ and hs-cTnI,^1^^–^^5^ which are closely related to the disease activity of COVID-19 in the literature. Therefore, we hypothesized that increased myocardial repolarization prolongation in patients with COVID-19 might be affected by disease activity and that arrhythmic events in these patients could be predicted in advance.

### Limitations

There are some important limitations to our study, including the retrospective design of the study and the number of patients enrolled. In addition, arrhythmic events and clinical follow-up parameters were not evaluated, due to the small number of patients and lack of clinical follow-up. Prospective studies with more patients can provide more meaningful information. In our study, medications and medical treatments that prolong QT were taken as exclusion criteria, but no genetic evaluation was performed for long or short QT. This hereditary channelopathy may not be very meaningful due to its rarity. Magnetic resonance imaging was not performed for cardiac involvement or myocarditis due to COVID-19. Another important limitation to our study is the inability to evaluate the effects of drugs such as hydroxychloroquine and azithromycin, which are frequently used to treat COVID-19, on ventricular repolarization.

## Conclusion

Although the main issue related to mortality and morbidity in patients with COVID-19 is acute lung disease, the available evidence indicates that one out of every five COVID-19 patients has myocardial damage. Our study showed that, in addition to previous COVID-19 studies in the literature, myocardial repolarization disorder occurred in addition to increased myocardial damage in patients with severe pneumonia. In patients with COVID-19, Tpe interval, Tpe/QT ratio, and Tpe/QTc ratio, which are among the dispersion of transmural ventricular repolarization parameters, were found to be increased, without prolonged QT and QTc intervals. This was more pronounced in patients with severe COVID-19 and severe pneumonia, and it may be associated with increased inflammation and myocardial damage. For patients with COVID-19, especially those with severe pneumonia, it should be kept in mind that prolongation may occur in ventricular repolarization. In this study, we cannot definitively conclude that the ECG changes observed are directly related to COVID-19 infection or inflammation, but rather associated with severe COVID-19 scenarios, which might involve other causes of inflammation and comorbidities.
